# Rejoinder in reply to critical appraisal on “factitious schizophrenia”

**DOI:** 10.4103/0019-5545.37675

**Published:** 2007

**Authors:** Rajnish Raj, Balwant Singh Sidhu

**Affiliations:** Department of Psychiatry, GMC and Rajindra Hospital, Patiala, Punjab, India

Sir,

We are grateful to the authors of “factitious schizophrenia”[1,2] for having opened the Pandora's box again. A case report is to be presented on the basis of facts, logic and reason. No statement is valid universally for all times. The case report and reply are gauged with many inconsistent premises, ambiguous and doubtful evidences, and they lack diagnostic validity of factitious disorder. The authors' argument in defense of their diagnosis is based on personal conjectures with bias entering in their results.

There are certain fallacies inherent in their deductive and inductive reasoning. The patient's initial contact with the psychiatrist for depression, transient blindness (para 4, page 170)[1] and schizophrenia (para 6, page 169)[1] was at the age of 16 years. The diagnosis of schizophrenia was confirmed by the psychiatrist in the Department of Psychiatry, Pgimer0 (post graduate institute of medical education and research, Chandigarh), and treated for more than a year with antipsychotic drugs, i.e., Risperdone and Ziprasidone; whence, the patient developed left-sided gynecomastia. There is self-contradictory explanation regarding antipsychotic medication, its initiation and continuation for two years (para 3, page 203; para 2, page 204).[2] Patient had developed seizure at the age of 17 years (para 4, page 170),[1] which later turned out to be pseudoseizure,[3] not so uncommon presentation; up to 10% of cases with seizures disorder have feigned symptoms (para 9, page 170).[1] At the age of 18 years, patient reported to psychiatric emergency for attempted suicide, atypical features of depression, hopelessness and continuous auditory hallucinations (para 5, page 169).[1] Hence, total duration of illness and treatment was two years (para 3, page 203).[2]

He was diagnosed as emotionally unstable personality, impulsive type on international personality disorder examination, IPDE (para 6, page 170).[1] On psychological analysis, it was stated that initially he was unaware of the symptoms, i.e., unintentional. A motivation to assume sick role was to save his passive mother from a dominating and anankastic (obsessive) father, who forced sexual acts against her wish (para 3, page 170)[1] - a primary gain; and continuation of symptoms was for care, love and affection - a secondary gain.

The patient's acceptance that he was feigning symptoms for the last two years and authors' contention that it was factitious disorder,[4] predominantly psychogenic type, might have taken place during therapeutic relationship. The issue of boundary violation by borderline personality disorder (BPD) patients is not uncommon. This is essentially true when the clinical picture is dominated by psychopathic traits (as described by Hare) of the intensely narcissistic type - grandiosity, conning, lack of remorse, lying and manipulative behavior.[5] The therapist might have been idealized by the patient during the session.

The authors' futile attempt to delineate a part of illness (factitious disorder) as disease and neglecting the whole[6] (borderline personality disorder) panorama of symptoms, i.e., atypical features of depression, self-harm, suicidal gestures, impulsivity, simulation psychosis, during stressful condition, pseudoseizure and dissociation was erroneous. The following points need reappraisal: there is considerable comorbidity between BPD and various dissociative symptoms, including depersonalization, derealization and loss of reality, which are not uncommon and may contribute to the psychotic-like symptoms that a patient with BPD may experience.[7–10]

Depression, often with atypical features, is particularly common in patients with BPD. Although the distinction can be difficult to make, depressive features that appear in BPD with a particular characteristic are emptiness, self-condemnation, abandonment fear, hopelessness, self-destructiveness and repeated suicidal gestures.[11–13]

Primary features of BPD are impulsive self-destructive behavior, substance abuse, risky behavior, self-mutilation and suicidal attempts. The behavior is thought to reflect the difficulties patients with BPD have with modulation and containment of intense emotions or impulses. They exhibit suspiciousness, referential thinking, paranoid ideation, illusion, derealization, depersonalization or hallucination-like symptoms.[14]

In the light of above evidences, the truth of borderline personality may get revealed with a pure rationalistic and scientific inquiry and it removes the veil of darkness from the confounding factor like factitious schizophrenia, which is one-ninth part of the diagnostic criteria for borderline personality disorder[14]; the truth is thus enlightening.
